# Characterization of Brown Seaweed (*Ascophyllum nodosum*) and Sugar Kelp (*Saccharina latissima*) Extracts Using Temporal Check-All-That-Apply

**DOI:** 10.3390/foods14152565

**Published:** 2025-07-22

**Authors:** Zach Adams, Nicoletta Faraone, Matthew B. McSweeney

**Affiliations:** 1 School of Nutrition and Dietetics, Acadia University, Wolfville, NS B4P 2R6, Canada; 0210969a@acadiau.ca; 2 Department of Chemistry, Acadia University, Wolfville, NS B4P 2R6, Canada; nicoletta.faraone@acadiau.ca

**Keywords:** macroalgae, seaweed, sensory perception, dynamic, consumer, temporal methods, HS-SPME, GC-MS

## Abstract

Seaweed is a sustainable ingredient that has been suggested to improve the nutritional aspects as well as the sensory properties of different food products. The objective of this study was to evaluate the flavor properties of extracts from brown seaweed (*Ascophyllum nodosum*) and sugar kelp (*Saccharina latissimi*) obtained at different temperatures. These varieties commonly grow in the Atlantic Ocean. The seaweed samples were extracted using water at three different temperatures (50 °C, 70 °C, and 90 °C). The volatile fraction of the extracts was extracted with headspace solid-phase microextraction and analyzed by gas chromatography–mass spectrometry. The headspace chemical composition varies significantly among seaweed extracts and at different extraction temperatures. Major classes of identified compounds were aldehydes, ketones, alcohols, hydrocarbons, and halogenated compounds. Extracts were also evaluated using temporal check-all-that-apply (with 84 untrained participants). The different temperatures had minimal impact on the flavour properties of the brown seaweed samples, but the extraction temperature did influence the properties of the sugar kelp samples. Increasing the extraction temperature seemed to lead to an increase in bitterness, savouriness, and earthy flavor, but future studies are needed to confirm this finding. This study continues the exploration of the flavor properties of seaweeds and identifies the dynamic flavor profile of brown seaweed and sugar kelp under different extraction conditions.

## 1. Introduction

Seaweed, or macroalgae, has garnered significant attention from researchers and the food industry due to its nutritional and functional properties [1]. Seaweeds are rich in bioactive compounds, including polysaccharides (e.g., alginate, carrageenan, and agar), proteins, polyunsaturated fatty acids, vitamins (notably A, C, E, and B12 [however, much of B12 quantified in assays is a B12 analog]), and essential minerals (e.g., iodine, calcium, and iron) [2]. Seaweed can be used as a potential functional ingredient to enhance the nutritional benefits of various food items. The dietary fibre content of seaweed can contribute to gastrointestinal health and glycemic control [3], and seaweed can also contain antioxidants and anti-inflammatory agents that may reduce the risk of chronic diseases [3]. Seaweed-derived hydrocolloids are commonly used in the food industry for various purposes, including texture modification, water retention, and shelf-life extension in processed foods [4]. From a sustainability perspective, seaweed cultivation requires no arable land or freshwater, offering an eco-friendly solution to meet growing global food demand [5]. Therefore, the use of seaweed in food may have a multifaceted benefit, including nutritional enhancement of foods, as well as improving the environmental sustainability of current food processing.

Consumers have also expressed interest in consuming seaweed [6,7]. However, for seaweed to be a viable ingredient for the food industry, the impact of extraction temperature on sensory properties needs to be explored. Furthermore, studies need to be conducted investigating the dynamic sensory perception of seaweeds. Studies have incorporated seaweed into a variety of different food items, including chocolate [8], noodles [9], bread [10], fish cakes [11], and crackers [12] (most of these studies have used static sensory methodologies). These studies have found varied degrees of consumer acceptance of seaweed addition to these products. Seaweeds are often described as having marine-like flavors, such as crustacean, fishy, and oceanic [13,14,15,16]. These flavors may result from the presence of dimethyl sulfide [17]. They have also been associated with savory (i.e., umami) and salty tastes [18,19]. Seaweeds are rich in glutamic acid and nucleotides, which contribute to an umami taste [20]. These flavor properties are acceptable to consumers when added to certain foods, but can also limit their use in other food items [21]. Some seaweed varieties may possess earthy, bitter, and herbal notes due to the presence of aldehydes and sulphur-containing substances [22]. Other varieties may be associated with sweetness due to the presence of amino acids, such as alanine and glycine [20]. Furthermore, the processing of seaweed can impact its flavor properties, with cooking or thermal processing reducing marine flavors [23], and fermentation can reduce flavors associated with dimethyl sulphide [24].

Traditionally consumed in Asia, dashi is a soup broth made from kombu seaweed (*Saccharina japonica*) [19,25]. Inspired by this dish, we aim to evaluate how this cooking process extracts different flavors from brown seaweed and sugar kelp, as these varieties are grown in Atlantic Canada [26,27]. Brown seaweed (*Ascophyllum nodosum*) is widely found along the North Atlantic coastlines [28]. It is rich in structurally diverse bioactives, such as alginates, laminarin, fucoidan, and polyphenols, and has been widely used in the food industry for its gelling, stabilizing, and thickening properties. Studies have been conducted to investigate the inclusion of brown seaweed in various food items [10,29,30], beyond its use as a hydrocolloid or gum. Most of these studies have stated that its inclusion is acceptable at low levels, but beyond that, it results in off-flavors and marine-like flavors [8,31]. Sugar kelp (*Saccharina latissima*) is native to the coastal waters of the Pacific and North Atlantic oceans [32]. Similar to other seaweed varieties, it has emerged as a potential food ingredient due to its nutritional composition, sustainable cultivation, and bioactive potential [33]. It has been described as having a mild umami taste with some sweetness and less marine-like flavors [34].

Before these seaweeds can be introduced to different food items, their flavor properties need to be more fully defined. This study utilized temporal check-all-that-apply (TCATA) and solid-phase microextraction (HS-SPME) to characterize their flavor properties of two varieties of seaweed. These methods allow for an objective evaluation of the seaweeds’ flavor (HS-SPME), as well as consumer perception using a dynamic sensory methodology (TCATA). TCATA extends the use of check-all-that-apply and allows for continuous selection (or deselection) of attributes based on their relevance to the sample [35]. During the TCATA task, participants are asked to place the sample in their mouths and immediately begin to evaluate their sensory perception of the sample, as well as how their perception changes over time [36]. In past studies, TCATA has been successfully used with untrained assessors or consumers and is easy for untrained assessors to complete [37]. TCATA is a rapid sensory method that provides a greater depth of information about the flavor properties of the seaweeds than static methods and how their flavors may change over the consumption period. Furthermore, the headspace chemical composition of the seaweed extracts evaluated using solid-phase microextraction (HS-SPME) coupled with gas chromatography–mass spectrometry (GC-MS) to assess the volatile organic compounds (VOCs) present [13].

The objective of the study is to evaluate the flavor properties of extracts from brown seaweed and sugar kelp produced at three different temperatures (50 °C, 70 °C, and 90 °C) using both TCATA and HS-SPME/GC-MS.

## 2. Materials and Methods

### 2.1. Samples

Both varieties of seaweed (sugar kelp and brown seaweed) were purchased from local companies (Nova Scotia, Canada), and all seaweed was collected off the coast of Nova Scotia, Canada. The water extraction method was followed as outlined by LaFeuille et al. [25] and inspired by the traditional method of making dashi [18]. Twenty grams of each seaweed was mixed with 1 L of tap water and then extracted at three different temperatures (50 °C, 70 °C, and 90 °C) for one hour. The water extracts were filtered and centrifuged (12,000× *g*) for twenty minutes at room temperature. Samples are labelled according to the seaweed species and extraction temperature. For example, BS (brown seaweed) and SK (sugar kelp) are reported as BS50 and SK50 when cooked at 50 °C.

### 2.2. SPME Seaweed Headspace Analysis

The extraction of VOCs from seaweed water extracts was performed according to Puspitasari et al. [38], LaFeuille et al. [25], and Vilar et al. [13] with some modifications. Headspace solid-phase microextraction (HS-SPME) analysis was performed by using a multiphase solid phase microextraction (SPME) 50/30 μm divinylbenzene/carboxen^TM^/polydimethylsiloxane (CAR/DVB/PDMS) (Agilent Technologies Ltd., Santa Clara, CA, USA) fibre of 80 μm thickness [13]. The fibre was conditioned before use according to the manufacturer’s instructions in the SPME Arrow Conditioning port at 200 °C for 10 min. Seaweed extract samples (5 mL) were transferred into 20 mL Precision Thread Headspace glass vials (previously washed in unscented soap and water, rinsed with acetone, and dried with a heat gun) with magnetic steel caps and PTFE/Sil (100 pk) vial septa (Agilent Technologies Ltd., Santa Clara, CA, USA). After equilibrating at 35 °C for 5 min under agitation, the SPME fibre was exposed to the seaweed headspace for 5 min and then desorbed in the GC injection port at 250 °C for 10 min under splitless conditions. The analysis of volatile compounds was performed using an Agilent 7890B gas chromatograph and an Agilent 5977B single quadrupole mass spectrometer (Agilent Technologies Ltd., Santa Clara, CA, USA) equipped with an HP-5ms Ultra Inert fused silica capillary column (30 m × 250 μm × 0.25 μm) (Agilent Technologies Ltd., Santa Clara, CA, USA) with the following parameters: ionization energy, 70 eV; mass signal acquisition of 30–350 amu (atomic mass unit); scan speed, 4.4 scans/s. The separation was achieved according to the oven temperature program, initially set at 40 °C and held for 3 min. The temperature was then raised to 180 °C at 10 °C/min and subsequently to 250 °C at 35 °C/min, where it was held for 1 min. Helium was used as the carrier gas at a flow rate of 1.2 mL/min. The volatile compounds were identified by comparing the retention indices and mass spectra from the NIST Mass Spectral Search Program (NIST/EPA/NIH EI Mass Spectral Library v. 2.4, 2020, Scion Instruments, Livingston, Scotland) and by comparing the retention times (RT) and mass spectrum fragmentations of chemical standards with corresponding peaks present.

### 2.3. Participants

Participants for the sensory trial were recruited from the Annapolis Valley, Nova Scotia, Canada, based on whether they were interested in consuming seaweed. All participants (n = 84; 50 females, 34 males; age ranged from 18 to 65 years; average age of 37.6 *±* 10.5) provided informed consent before participating in the sensory trial.

### 2.4. Sensory Procedure

The study was conducted in accordance with the Declaration of Helsinki and approved by the Acadia University Research Ethics Board (#13-72; 15 January 2025). The TCATA method was verbally explained to the participants, and then sensory properties included in the TCATA questions were shown to the participants. Any questions about the method or the sensory properties were answered by the researchers. The participants then entered the sensory booths, and the questionnaire was presented using Compusense (Compusense Inc. Guelph, Ontario, Canada) on an iPad. The participants received 150 mL of each sample (at room temperature in clear plastic cups). Each sample was blinded with three-digit codes and presented following a completely randomized design. The participants were instructed to select all the sensory properties from the list that applied to the perception of the sample at each moment of the evaluation. The attributes included were salty, fishy, bitter, savory, earthy and seafood/crustacean. The attributes were included based on a literature review [14,23,34,39,40,41]. The attributes included in the TCATA question were reviewed by research assistants experienced in sensory analysis (n = 7) to ensure relevance to the samples. To begin, the participants were asked to click the start button and at the same time take a sip (a regular mouthful) of the sample. They were then asked to keep the sample in their mouth and then swallow the sample after ten seconds and take another sip. The participants were instructed to take five sips throughout the evaluation. The participants were asked to evaluate the sample through the different sips and swallows. The evaluation period lasted ninety seconds, and they were informed that each attribute has a fading time of eight seconds. They were instructed to reselect the attribute if it was still relevant to their perception of the sample. There was a two-minute break period where participants were asked to drink water to cleanse their palate. After evaluating all of the samples, the participants then completed demographic questions.

### 2.5. Statistical Analysis

The TCATA data were analyzed as outlined by the procedure by Castura et al. [9]. Aggregated data from all the participants were visualized using line plots, and the average citation proportion was smoothed using a cubic smoothing spline and plotted as 0.1-s increments. As described by McMahon et al. [42], the analysis of the average proportion was calculated. Differences in the average proportions for each attribute for each sample were compared using an analysis of variance and Tukey’s Honest significance difference test (95% confidence). Difference curves were generated using Compusense software to evaluate differences in the flavor properties based on the extraction procedure. Descriptive statistics evaluated the demographic questions.

## 3. Results

### 3.1. Analysis of Volatile Compounds 

The VOCs from brown seaweed and sugar kelp extracted at different temperatures are presented in Table 1. Overall, the VOC composition of the two different seaweed varieties varies significantly. The VOCs identified have previously been identified in seaweeds [13,25,38], and include many earthy, green, and citrus aromas associated mainly with the presence of aldehydes (e.g., 2-hexenal, heptanal, octanal, nonanal), followed by hydrocarbons (e.g., pentadecane), halogenated compounds (pentane-1-iodo, heptane-1-iodo, octane-1-iodo), ketones (e.g., trans-beta-ionone, 6-octen-2-one), and alcohols (e.g., 2-octen-1-ol). Terpinolene and heptane-1-iodo were present in the brown seaweed extracted at a temperature of 50 °C, however, they are not detected anymore in the extracts at higher temperatures. Surprisingly, the kelp extracted at 90 °C contained more VOCs when compared to the extracts from lower temperatures, indicating that high-temperature extraction conditions promote the release of smaller VOCs such as hexanal, 2-hexenal, heptanal, and benzaldehyde.

When comparing the two different varieties of seaweed, the brown seaweed samples exhibited many green, fatty, and waxy VOCs, while the sugar kelp is associated with sweeter and herbal aromas. Hexanal (i.e., green, fruity), D-limonene (i.e., lemon, orange), nonanal (i.e., rose, orange), and decanal (i.e., fatty, citrus) were detected in all extracted samples from both varieties at different temperatures.

### 3.2. TCATA

**Figure 1:**
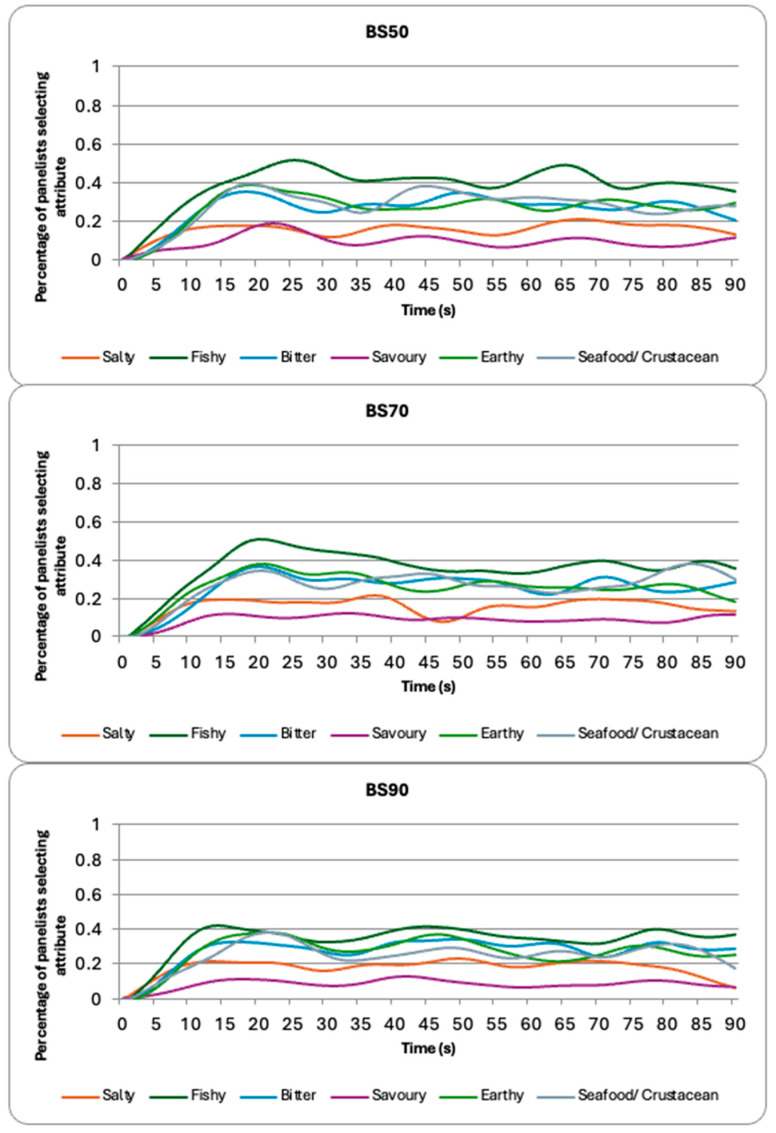
TCATA curves for the brown seaweed samples cooked at the three different temperatures (50 °C, 70 °C, and 90 °C).

**Figure 2:**
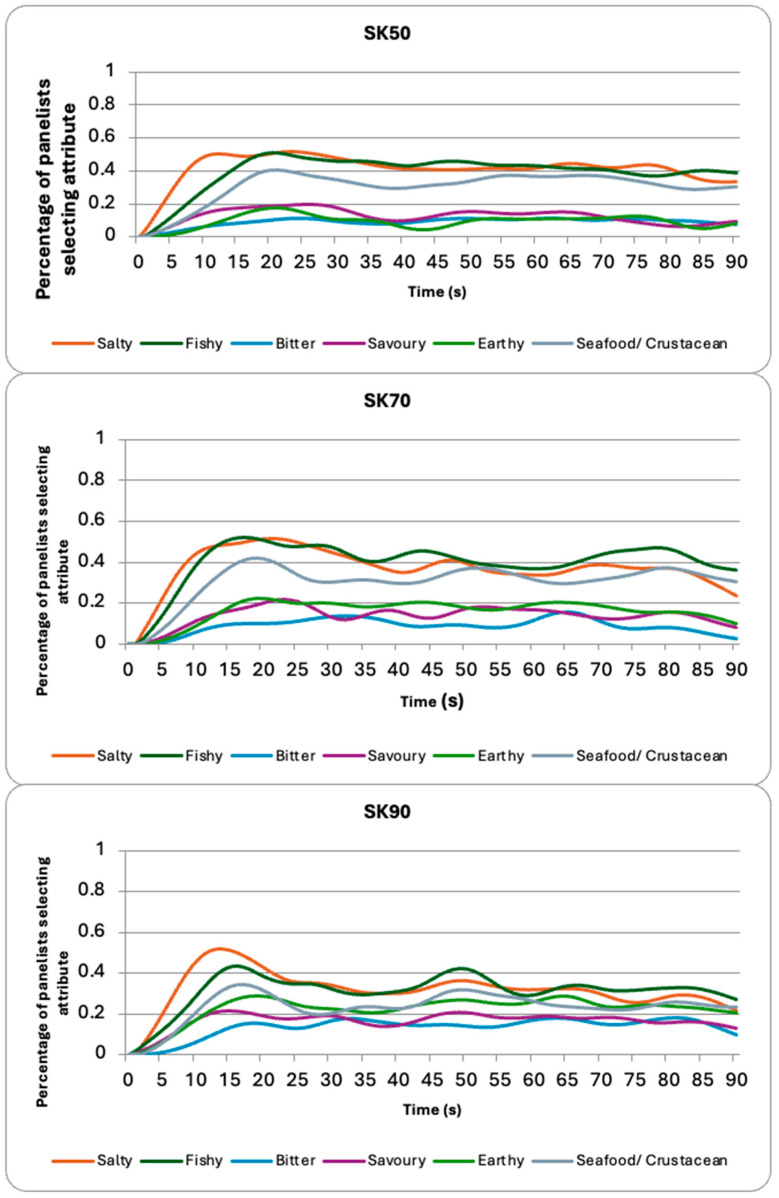
TCATA curves for the sugar kelp samples cooked at the three different temperatures (50 °C, 70 °C, and 90 °C).

The next part of the study was to evaluate whether consumers could identify these differences in the seaweed varieties and extraction times. Plots outlined the proportion of citations for the brown seaweed samples (Figure 1) and sugar kelp samples (Figure 2) for each sensory attribute throughout the ninety seconds of evaluation. The average proportions of citations are also shown in Table 2. The brown seaweed samples were perceived by the participants to have high levels of fishy and earthy attributes. Furthermore, they were identified as being bitter and having a seafood or crustacean flavor. The BS50 sample had a significantly higher perception of fishy attributes than the BS90 sample (*p*-value = 0.029; Table 2).

The sugar kelp samples (Figure 2) were perceived as salty, but participants also described them as having a fishy and seafood/crustacean flavor. These other three attributes (e.g., savory, bitter, and earthy) were selected less often by the participants. The temperature used for extraction had a direct impact on the perception of saltiness: as the temperature increased, the perception of saltiness decreased. SK50 was found perceived to be significantly saltier than the SK70 (*p*-value = 0.005) and SK90 (*p*-value < 0.0001) samples (Table 2). However, SK90 was also found to be significantly more bitter (*p*-values = 0.001 (SK50) and <0.0001 (SK70)) and earthy (*p*-values < 0.0001 (SK50) and <0.0001 (SK70)) than the other sugar kelp samples. The SK90 was also found to have the least amount of seafood/crustacean (*p*-values < 0.0001 (SK50) and <0.0001 (SK70)) and fishy flavor (*p*-values < 0.0001 (SK50) and <0.0001 (SK70)) in comparison to the other sugar kelp samples.

**Figure 3:**
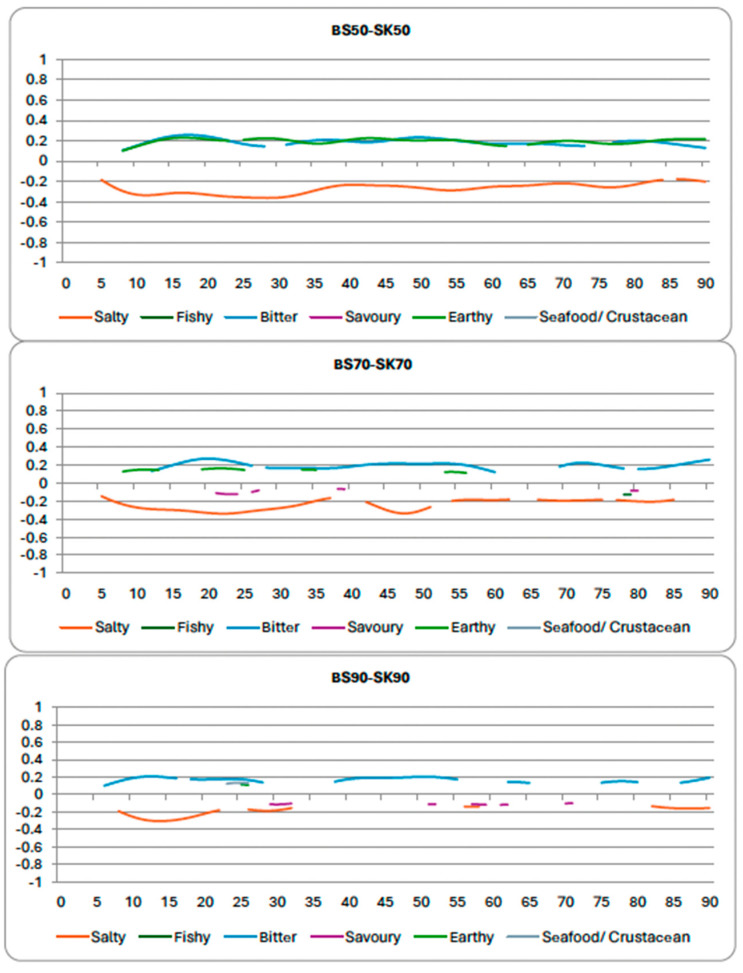
Difference curves for the brown seaweed in comparison to sugar kelp samples cooked at the same temperature (first plot = 50 °C; second plot = 70 °C; and third plot = 90 °C).

The average proportions (Table 2) and the difference curves (Figure 3) illustrate the differences between the two seaweeds. When looking at the average proportions of citations (Table 2), the sugar kelp samples were significantly higher in saltiness (*p*-values < 0.0001) and savoriness (*p*-values < 0.0001) than the brown seaweed samples (at all temperatures of extraction). The brown seaweed samples were found to be significantly more bitter (*p*-values < 0.0001) and earthy (*p*-values < 0.0001) than all sugar kelp samples (*p* < 0.05). At 50 °C and 70 °C extraction temperatures, the sugar kelp sample was found to have significantly more seafood/crustacean flavor (*p*-values = 0.040 and 0.030, respectively), but this result was not found when the seaweed was extracted at 90 °C. In both seaweeds, the amount of fishy flavor decreased with increased extraction temperature.

## 4. Discussion

The VOCs identified in the different seaweed varieties have been reported in previous studies conducted on other seaweed species [17,25,44]. Brown seaweed has been identified to have many unsaturated fatty acid derivatives (e.g., 2-nonenal, (E), and 2-octenal, (E)), and these compounds are known to lead to green, fatty aromas with some fruity aromas [25], agreeing with the results of this study. Furthermore, past studies investigating water extracts of *S. longicruris* (*latissimi*) or sugar kelp have identified that they possess honey, fruity, floral, and herbal aromas [14,25].

For the brown seaweeds, there was not much influence of the extraction temperature on the VOCs identified (Table 1) as hexanal, pentane 1-iodo, octanal, o-cymene, D-limonene, 2-octenal (E), 2-nonenal (E), nonanal, decanal, octane 1-iodo, 2-decenal (E), 2,4-decadienal (E,Z), and pentadecane found in BS50, BS70 and BS90. The only changes in VOCs are the presence of terpinolene (sweet, piney, oily) and heptane 1-iodo (slightly sweet and oily) in the BS50 extract. These compounds all contribute to the grassy, fruity, and fatty aromas that have been identified in past studies on brown seaweed [13]. Heat is known to alter the VOCs present in foods and seaweeds [45], but it seemed to only have a minor impact in this study. This may be due to the environment in which the seaweed was grown. In another study, the aqueous extract of the seaweed *Palmaria palmata* was found to have the most effective extraction parameter at the lowest temperature [25]. This may also be the case in our study, as the BS50 was associated with more VOCs than the samples extracted at higher temperatures (BS70 and BS90).

In contrast, the sugar kelp’s VOCs were impacted drastically by the different temperatures. Hexanal, D-limonene, cyclohexanone 2,2,6-trimethyl, nonanal, decanal, 2-6-octadine 2,6-dimethyl, furan 2-pentyl, and trans-beta-ionone were present in all extracted sugar kelp samples. However, at elevated temperatures, terpinolene was not present, and neither was 1,6-dimethylhepta-1,3,5-triene. Terpinolene (sweet, piney, oily) was also only present in the BS50. The elevated temperatures (in samples SK70 and SK90) led to different VOCs being present in the extracted sugar kelp samples, including 2-hexenal (E), heptanal, pentane 1-iodo, benzaldehyde, 2-octen-1-ol (Z), octanal, 2-octenal (E), 2-nonenal (E), and alpha-fenchene. The heat treatment had a more prominent impact on the VOCs of the sugar kelp.

The impact of differing heat treatments on the sensory perception of the key tastes and flavors associated with seaweed was investigated. The brown seaweed was identified as having a bitter, fishy, earthy flavor with a seafood/crustacean aroma (Figure 1 and Table 2). These sensory properties have been identified in past studies on brown seaweed [8,41,46]. All these sensory properties were present throughout the 90-s evaluation by the participants. Furthermore, seaweeds have been described as salty and savory, but in this study saltiness and savoriness were perceived less often than the other attributes included in the TCATA. This may present problems for studies that suggest seaweed could be used as a salt replacer or saltiness enhancer [39,47], if they introduce other flavors (fishy, earthy, seafood/crustacean flavor) and tastes (bitter). However, it must be acknowledged that the samples evaluated were experimental aqueous extracts and would not be served like this in the real world. Future studies should continue to evaluate seaweed use as a salt replacer or saltiness enhancer in different food matrices. Similar to the identified VOC content, the different brown seaweeds did not differ in the average proportion of citation for the different sensory attributes; except the BS90 sample was identified to be significantly less fishy than the BS50 sample.

The sugar kelp samples were dominated by a salty taste, while also being associated with fishy and seafood/crustacean flavor. Saltiness has been identified in previous studies on consumer perception of sugar kelp; however, as noted above, the presence of additional flavors such as seafood, crustacean, and fishy notes may limit its suitability as a salt replacer. The other attributes (i.e., bitterness, savory, earthy) were selected less often by the participants. The extraction temperature had a significant impact on consumer perception of the sugar kelp, with the average citation of saltiness being significantly lower for the SK90 sample than for the SK50 and SK70 samples. The intensity of fishy and seafood/crustacean flavors also decreased significantly. The decrease in these attributes may be due to the significant increase in the other attributes (i.e., bitter, savory, earthy), which all increased substantially as the temperature rose. This result could be due to increased flavor complexity, which can reduce consumers’ perception of specific attributes [48]. Furthermore, increased sensory complexity or flavor complexity can lead to an increased number of concurrently perceived sensations, which in turn can lead to delayed answers during the TCATA task [49,50]. However, the overall flavor complexity was not explored in this study and should be investigated in future studies.

The increase in these flavors may be linked to the presence of different VOCs in the SK70 and SK90 samples as discussed above. Many of these VOCs are identified as having green and earthy aromas (e.g., 6-octen-1-ol and 2-octen-1-ol, (Z)), which could have contributed to the increased perception of the earthy attribute by the participants. Furthermore, bitterness is often associated with earthy foods by consumers, as many of these foods are also naturally bitter. The presence of heptanal in the sugar kelp samples extracted at an elevated temperature may have also contributed to an increased perception of bitterness by the participants. Heptanal has been found to have a pungent aroma, which can be associated with bitterness in consumers’ minds [51]. Trained panelists are often able to differentiate the pungency of heptanal from the bitter attributes, but that was not observed in this study. This result may be due to use of untrained participants in this study, which is a limitation. Additionally, the SK70 and SK90 samples were found to contain benzaldehyde, which has a bitter almond aroma, and this may have influenced the perception of bitterness [43].

Comparing the two different seaweed varieties illustrates the differences between the samples. The sugar kelp samples were all found to be significantly higher in saltiness and savoriness than the brown seaweed samples (clearly illustrated by the difference curves). The brown seaweed samples were significantly more bitter than the sugar kelp samples. To the best of our knowledge, there are no studies that have compared the sensory properties of these two seaweed varieties, although some have examined their nutritional profiles (e.g., [52]). This study suggests that sugar kelp may be more suitable than brown seaweed when saltiness and savoriness are desired in the final product. However, there is a caveat of growing location that must be considered, as it can influence the sensory properties [25]. Growing location was not compared in this study (both varieties harvested from Nova Scotia, Canada), but future studies should investigate geographic variability. Both seaweed varieties showed an inverse relationship between temperature and the perception of fishy and seafood/crustacean flavor. Heat treatment may reduce these off-flavors, but it also increases the participants’ perception of earthiness and bitterness in the sugar kelp samples.

This study evaluated how water heat treatment can influence the flavor properties of two seaweed varieties; however, many other seaweed varieties also need to be investigated. Studies primarily need to be employed using sensory methodologies to investigate consumers’ perceptions of these sustainable ingredients. Furthermore, the use of trained panelists to identify small differences in the flavors of seaweed would be ideal. Future studies could also use gas chromatography-olfactometry to further analyze the aroma compounds of these seaweed varieties and others. Future studies comparing brown seaweed and sugar kelp grown in different environmental conditions and geographic regions could provide a deeper understanding of their influence on the sensory properties. Consumers with an interest in seaweed consumption were recruited; however, exclusively selecting regular seaweed consumers may influence the results. Familiarity with a food item can impact sensory perception [53]. As stated above, the samples evaluated in this study were experimental. They would probably not be served in this form in a real-life situation. Also, the seaweeds were extracted using tap water as specified in previous studies [18,25], but future studies may want to use demineralized water. Still, future studies should evaluate the acceptability of seaweed extracted at different temperatures.

## 5. Conclusions

A few studies have investigated the VOCs of different seaweed varieties extracted from various sources. Still, this study builds on those past studies by employing dynamic sensory methodologies to examine how different extraction temperatures affect the sensory profile and consumer perception. The different extraction temperatures had a more profound impact on the flavor properties of the sugar kelp than the brown seaweed evaluated in this study. The sugar kelp samples at low extraction temperatures were associated with saltiness, fishy, and seafood/crustacean flavor. As the extraction temperature increased, bitterness, savoriness, and earthiness increased. The brown seaweed extracted at all temperatures was identified as bitter, with fishy, earthy, and seafood/crustacean flavors. This study continues the exploration of the flavor properties of seaweeds using both scientific assessments and human evaluations. These results aim to support the development of new seaweed products by providing insight into how different extraction temperatures affect the flavor properties of brown seaweed and sugar kelp.

## Figures and Tables

**Table 1:** Volatile organic compounds detected in the headspace of seaweed extracts (sugar kelp and brown seaweed varieties). Extraction was performed at different temperatures (50 °C, 70 °C, and 90 °C). Chemical identity (ID) is confirmed either by comparison with the retention time (RT) of chemical standards (S) or by the highest percentage match probability in the National Institute of Standards and Technology (NIST) database. Grey areas indicate presence, while white areas indicate absence.

Chemicals	RT	Brown			Sugar Kelp			ID	Odours ^1^
		50 °C	70 °C	90 °C	50 °C	70 °C	90 °C		
hexanal	5.015							S	Green, fruity
2-hexenal, (E)	6.128							S	Green, fruity
heptanal	7.056							S	Fatty, pungent
pentane, 1-iodo	7.406							NIST	Petroleum
2-heptenal, (Z)	8.075							NIST	Pungent, green
benzaldehyde	8.151							S	Bitter almond
2-octen-1-ol, (Z)	8.490							NIST	Fatty, green, nutty
6-octen-2-one	8.623							NIST	Green, earthy
furan, 2-pentyl	8.728							NIST	Earthy, vegetable
octanal	8.915							S	Fatty, citrus
o-cymene	9.315							S	Citrus
D-limonene	9.387							S	Lemon, orange
cyclohexanone, 2,2,6-trimethyl	9.508							NIST	Sweet, honey, tobacco
2-octenal, (E)	9.877							NIST	Green, cognac
terpinolene	10.368							S	Sweet, piney, oily
nonanal	10.638							S	Rose, orange
heptane, 1-iodo	11.075							NIST	Slightly sweet, oily
2-nonenal, (E)	11.512							NIST	Orris, waxy
1,6-dimethylhepta-1,3,5-triene	12.029							NIST	None
decanal	12.204							S	Fatty, citrus
octane, 1-iodo	12.676							NIST	Green
2-decenal, (E)	13.037							S	Waxy, fatty
2,4-decadienal, (E,Z)	13.497							S	Fatty, chicken-like, citrus-like
2,6-octadiene, 2,6-dimethyl	14.284							NIST	Citrus-like, tea
alpha-fenchene	14.698							NIST	Herbal, sweet, woody
trans -beta-ionone	16.113							S	Floral, woody, fruity
pentadecane	16.17							S	Waxy

^1^ Based on Burdock [43].

**Table 2:** The average proportion of the participants’ citations from the sensory properties included in the TCATA.

Sample	Salty	Fishy	Bitter	Savory	Earthy	Seafood/Crustacean
BS50	0.159 d ^1^	0.390 a	0.265 a	0.094 c	0.271 a	0.279 ab
BS70	0.154 d	0.357 ab	0.257 a	0.088 c	0.260 a	0.264 bc
BS90	0.180 d	0.342 b	0.276 a	0.084 c	0.269 a	0.252 bc
SK50	0.417 a	0.391 a	0.091 c	0.125 b	0.090 d	0.304 a
SK70	0.373 b	0.402 a	0.087 c	0.161 c	0.161 c	0.306 a
SK90	0.321 c	0.314 b	0.133 b	0.164 c	0.221 b	0.233 c

^1^ Means in the same column, with the same letter, are not significantly different (95% confidence interval).

## Data Availability

The original contributions presented in the study are included in the article, further inquiries can be directed to the corresponding author.
